# Investigation of Anti-Infection Mechanism of Lactoferricin and Splunc-1

**DOI:** 10.1155/2014/907028

**Published:** 2014-04-30

**Authors:** Yung An Tsou, Hung-Jin Huang, Wesley Wen Yang Lin, Calvin Yu-Chian Chen

**Affiliations:** ^1^Otolaryngology Head and Neck Surgery, China Medical University Hospital, Taichung 40402, Taiwan; ^2^School of Medicine, College of Medicine, China Medical University, Taichung 40402, Taiwan; ^3^Department of Chinese Pharmaceutical Sciences and Chinese Medicine Resources, College of Pharmacy, China Medical University, Taichung 40402, Taiwan; ^4^Department of Biological Science and Technology, National Chiao Tung University, Hsinchu 30010, Taiwan; ^5^Department of Biomedical Informatics, Asia University, Taichung 41354, Taiwan

## Abstract

The innate immune system is the first line in the defense system and prevents the body from further bacteria, virus, or fungal infections. Most of the innate immune system is relevant to mucosa immunity. Lactotransferrin is secreted from the human mammal breast duct epithelial tissue and strengthens infant immunity to defense with regard to outward pathogens. Splunc-1 is also an innate material secreted from the soft palate, lung, nasal cavity epithelium, and mucosa. It helps with mucosa defense against bacterial, virus, and even fungus. LPS is the main etiology of Gram-negative bacilla infection source. And studies of lactoferricin and slpunc-1 both can combine with LPS and subsequently cause insults to the mucosa. Although, we know that both of them partake in an important role in innate immunity, we do not know the effects when they work together. In this study, we just overview silicon stimulation to examine the combination of Lactoferricin and Splunc-1 and the effect with regard to LPS.

## 1. Introduction 

Splunc-1 is secreted by the soft palate, lung, and nasal cavity epithelium and is considered an innate material to help mucosa immunity [1]. There have been a number of studies which have proven the antibacterial benefits [2] with regard to* Staphylococcus aureus*,* Streptococcus*,* Pseudomonas* [3, 4], mycoplasma pneumonia [5], and klebsiella pneumonia [6]. It is also considered to have antibiofilm functionality through a reduction of the surface tension by regulating the airway surface liquid volume [7, 8]. It is also very important connector to coordinate innate and adaptive immunity [9]. Furthermore, it is quite important to protect the upper airway from infections [10] and to neutralize pathogens containing lipopolysaccharides (LPS).

LPS is the major component of Gram-negative bacteria (GNB) outer membrane and it belongs to lipoglycans, comprising a lipid and a polysaccharide connected by a covalent bond. Many kinds of GNB have this crucial pathogenic material and lead to human infections.

Lactoferricin is an iron-binding glycoprotein not only found in milk or human mammal ducts but also found in exocrine secretions such as nasal secretions, tears, saliva, urine, uterine secretions, and amniotic fluids and is considered a crucial innate immune material [11]. Bovine lactoferricin has even stronger antimicrobial function [12]. We examined the cyclic domain of short derivatives within Bovine lactoferricin, which has the power to protect against* E. coli*,* S. aureus*, and* C. albicans* [13]. It can also bind to LPS and reduce the inflammation and infection condition via inhibition of IL-1 and LPS activity in the intervertebral disc [14].

In addition, bactericidal effects were found in lactoferricin material by their binding ability to LPS in Gram-negative bacteria and stabilized cell membranes [15, 16]. The upper airway (nasal sinus) biofilm formation is also correlated to a reduced level of lactoferricin [17]. Chronic rhinosinusitis and infection correlate not only to decreased lactoferricin but also to decreased Splunc-1 expression [18].

**Figure 1:**
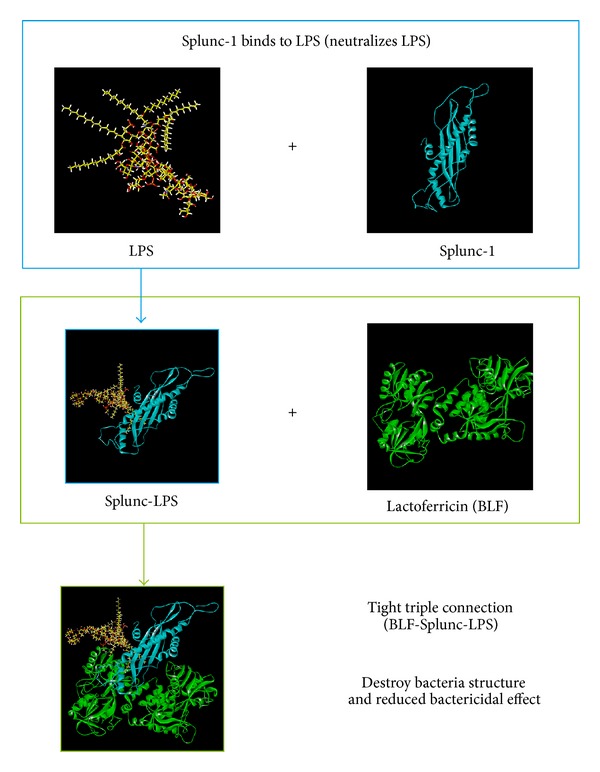
Study hypothesis.

Since these new silicon findings can help pharmaceutical companies to develop innovative drugs to reduce the biofilm formation in the airway to reduce further comorbidities by outward human pathogens [19], therefor, we used computer-aided drugs design according to many research for further investigation [20–24].* In silico* drug design includes database virtual screening [25–30], quantitative structure-activity relationship [31–34], protein-protein interactions [35], and molecular dynamics simulation [36–39], and these approaches are based on theory [40], web server [41], compound database [42], and risk targets [43–47] to perform studies of drug design or identify mechanism. Since both lactoferricin and Splunc-1 are important to protect human from LPS related infection, we hypothesize that the two proteins maybe combine to treat LPS related upper airway infections and might have synergistic effects to prevent human upper airway infection diseases as intractable sinusitis, tonsillitis, and severe pneumonia. (Figure 1). Therefore, we can potentially invent new drugs or nasal sprays to send these two important innate immune materials to the nasal cavity by nasal spray and send them to the lower airway by inhaler.

## 2. Materials and Methods

### 2.1. Structure Preparation

The protein structure of Splunc-1 was obtained from UniProt database; we used an I-TASSER server [48–50] (iterative threading assembly refinement algorithm) to build the 3D structure of Splunc-1 protein from the amino acid sequence. We utilized Ramachandran plot [51] and 3D profile [52] to validate the 3D structure of Splunc-1. We choose the most reliable prediction structure for further protein-protein interaction survey. X-ray crystal structure of Bovine lactoferricin was achieved from the PDB database (PDB:1BLF) [53]. 2D structure of LPS is gained from PubChem Compound database. We utilized MM2 force field [54] of ChemBioOffice 2010 software to optimize and calculate the 3D compound structure.

### 2.2. ZDOCK and LibDock Analysis

ZDOCK [55] and LibDock [56] programs in the Accelrys Discovery Studio 2.5.5.9350 (DS 2.5) [52] were used for protein and compound interaction. ZDOCK was employed to simulate Splunc-1 and Bovine lactoferricin interaction. Angular step size was set as 6 to analyze 54,000 possible proteins interaction poses; ZDOCK score was calculated by ZDOCK program, according to the shape complementarity of the protein complexes. LibDock is used to build protein-ligand interaction. The possible connecting methods of Splunc-1-lactoferricin complex to LPS are analyzed by LibDock program. We used the sphere of 40 Å radiuses as the binding area; polar and nonpolar molecules are regarded as hotspots for active site definition. The number of hotspots was set as 100 for conformer matching. The conformation method was using FAST mode. The final predicted protein-ligand complex was optimized by energy minimization, and the mode of minimization was set as smart minimization. CHARMM [57] force field was used for calculation in energy minimization.

### 2.3. Molecular Dynamics Simulations

The molecular dynamics simulation for protein-ligand complexes is analyzed by Standard Dynamics Cascade of DS 2.5 [52]. Energy minimization process is performed using two stages of minimizations by steepest descent and conjugate gradient [58, 59], and we do heating stimulation and set the system temperature increasing from 50 K to 310 K in 50 ps and then maintain in 310 K for 200 ps. Afterward, we performed constant temperature dynamics environment for 40 ns (NVT type) for production simulation. We set the decay time for temperature coupling as 0.4. Trajectory analysis for conformations changes survey is done by analyzing production simulation saved data by every 10,000 ps. The distance-dependent dielectrics method was utilized for solvent model. The time period for each simulation step was set as 2 fs. We constrain all bonds containing hydrogen by employing SHAKE algorithm [60] in the system during simulation.

## 3. Results

**Figure 2:**
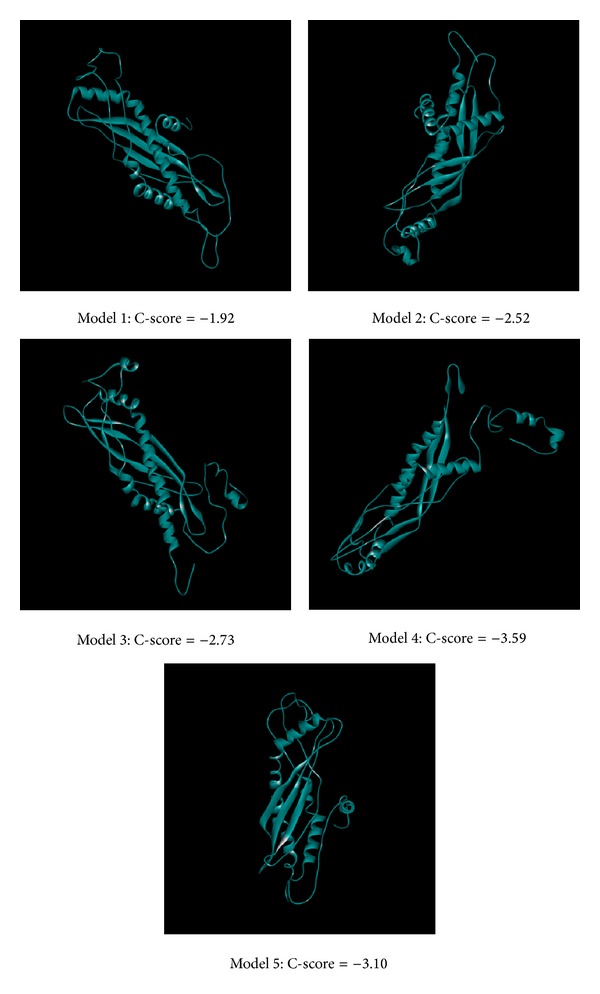
Five structures stimulated by I-TASSER; C-score shows that the reasonable ranges involved scores from −5 to 2, and the higher score renders a more reliable structure.

**Figure 3:**
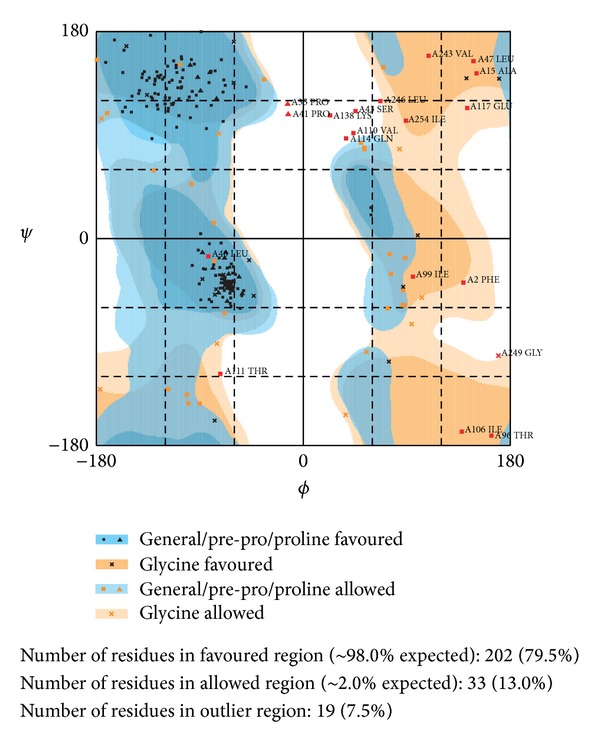
Ramachandran plot of Splunc-1 residues for structure validation, 79.5% and 13.0% residues in favored and allowed regions, respectively.

**Figure 4:**
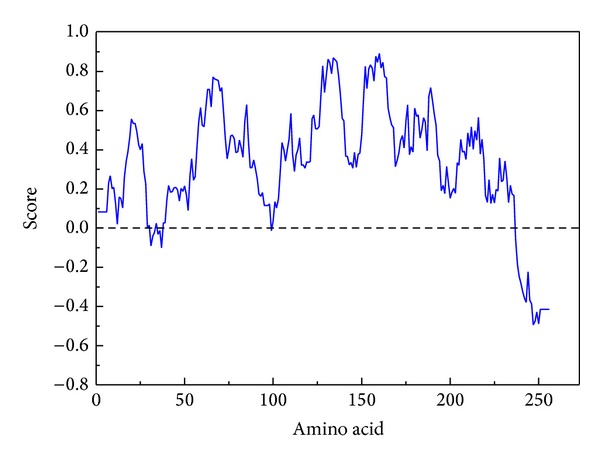
3D-profile analysis of SPLUNC residues; the verified score above zero indicated valid residues.

The amino acid is used for predicting possible five protein structures by I-TASSER server (Figure 2); all the C-score numbers are in the reasonable range; the higher score rendered more reliable protein structure. Then we validated the simulated Splunc-1 structural residues in their docking regions by Ramachandran plot, and 92.5% were distributed at a reliable region which was 79.5% and 13.0% of residues in favored and allowed regions, respectively, as shown in Figure 3. In Figure 4 we showed Splunc-1 residues by 3D-profile analysis, and valid residues were proven by most of the validation scores being above zero.

**Figure 5:**
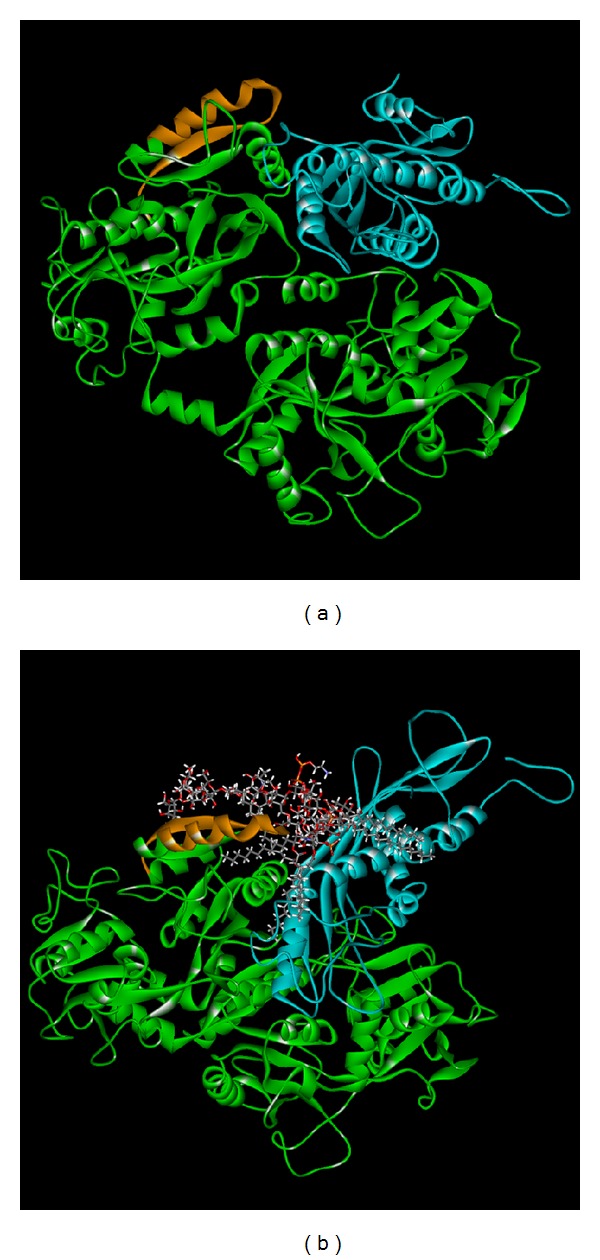
(a) The results of the connection of Splunc-1 (blue) and Bovine lactoferricin (green) (Pose 6 from ZDOCK); the orange part refers to the Bovine lactoferricin. (b) By the LibDock result, the Splunc-1 and Bovine lactoferricin complex could connect to the LPS (LibDock score = 180.368).

**Figure 6:**
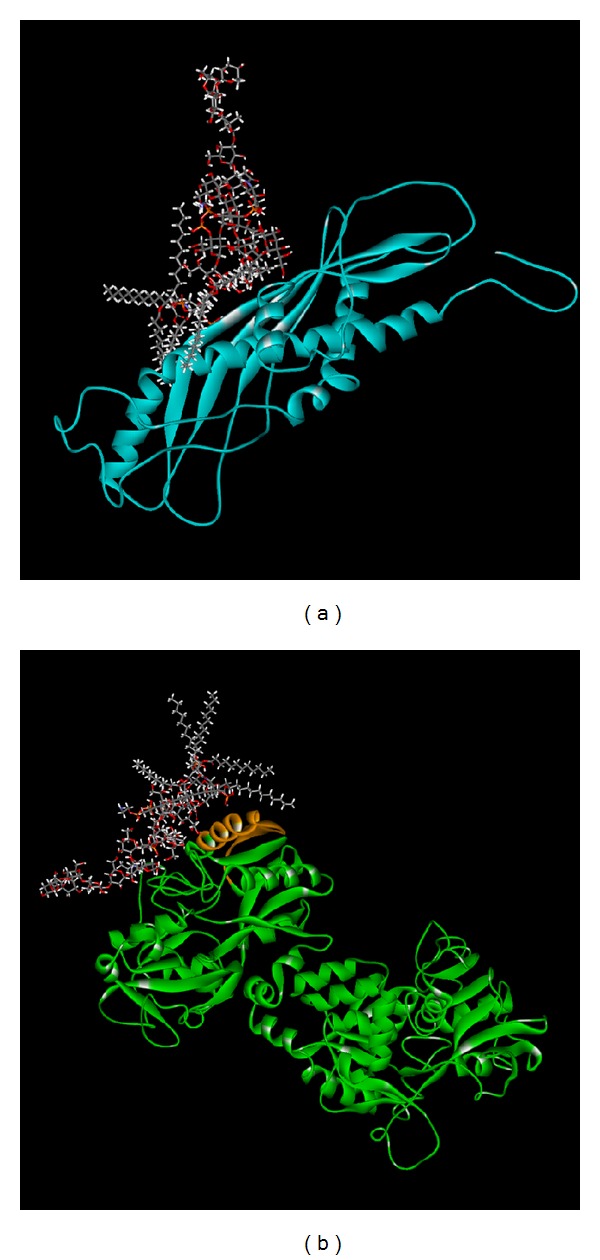
(a) Splunc-1 and (b) Bovine lactoferricin and their connected pose to LPS. The LibDock scores were 128.051 and 64.152.

Therefore, the connection of Bovine lactoferricin and LPS was based upon Model 1 structure for further research and poses survey. We further analyzed interactions between LPS and Splunc-1-Bovine lactoferricin complex by ZDOCK. We choose the top ten docking score for LPS binding for Splunc-1-Bovine lactoferricin complex (Table 1). We found that the LPS could be connected to the Splunc-1-Bovine lactoferricin complex (Pose 6) by LibDock and the LibDock score was 180.368 (Figure 5). Prior study indicated that Splunc-1-Bovine lactoferricin complex could connect to LPS, and we also proved that Splunc-1 and Bovine lactoferricin could connect to the LPS under LibDock program. And the LibDock score was 128.051 for PLUNC and LPS interaction and 64.152 for lactoferricin and LPS interaction (Figure 6). In addition, we found the Splunc-1 and Bovine lactoferricin complex indeed does have synergistic effect in connecting LPS. In further study, MD simulation (molecular dynamics simulation) in DS 2.5 was carried out to simulate the protein-protein and protein-ligand complexes. In addition, the stability of the binding poses in dynamic conditions was analyzed.

## 4. Discussion 

The upper airway organs including the nasal cavity, nasal sinus, pharyngeal mucosa, palatine, and lingual tonsils are the first defense organs that prevent humans from suffering further damage from bacteria, virus, and fungus and upper airway mucosa is also considered a part of innate immunity, especially regarding pathogens comprising LPS [61]. Lactoferricin and Splunc-1 are two very crucial materials for innate immunity secreted from the mammal glandular epithelium and upper airway [1–4, 11, 14, 62].

Persistent sinusitis related to biofilm formation also revealed low levels of lactoferricin and Splunc-1 [4, 17, 18]. Low levels of lactoferricin in the sinus mucosa area are more susceptible to biofilm formation [17]. The pathogen* Pseudomonas* and* Staphylococcus aureus* harbored in the human nasal sinuses are considered to be correlated to sinus biofilm formation and also showed a decreased Splunc-1 expression [63]. Chronic rhinosinusitis related to biofilm formation is very difficult to treat with penicillin and some studies have shown lactoferricin could be helpful and that it has a synergic effect with regard to penicillin resistant* Pseudomonas* infection [64]. Therefore, combine use innate immune materials as lactoferricin and Splunc-1 may partake in an important role for eradicating biofilm formation and preventing patients from further surgical treatment for intractable sinusitis or tonsillitis.

This is also worthy to be investigated for treating the human infection diseases related to biofilm in the future.

Although there have been studies concerning lactoferricin and Splunc-1 being used to suppress LPS related infection [2, 14, 61], there have been no studies concerning combining the two innate materials to combat LPS related infections. We found the two materials could both bind to LPS and form more stabilized compounds binding to LPS. In Figure 2, we selected the possible structure for Splunc-1 for further docking analysis and their docking power was also shown in Table 1. Then the docking of Splunc-1 to LPS and the docking of Lactoferricin, Splunc-1, and LPS are shown in Figure 5 and these three could be bound well in silicon simulation. In addition, the two innate immune materials could bind to LPS separately well as seen in Figure 6; each of them has good binding ability to LPS and this has also been proven in many human studies.

**Figure 7:**
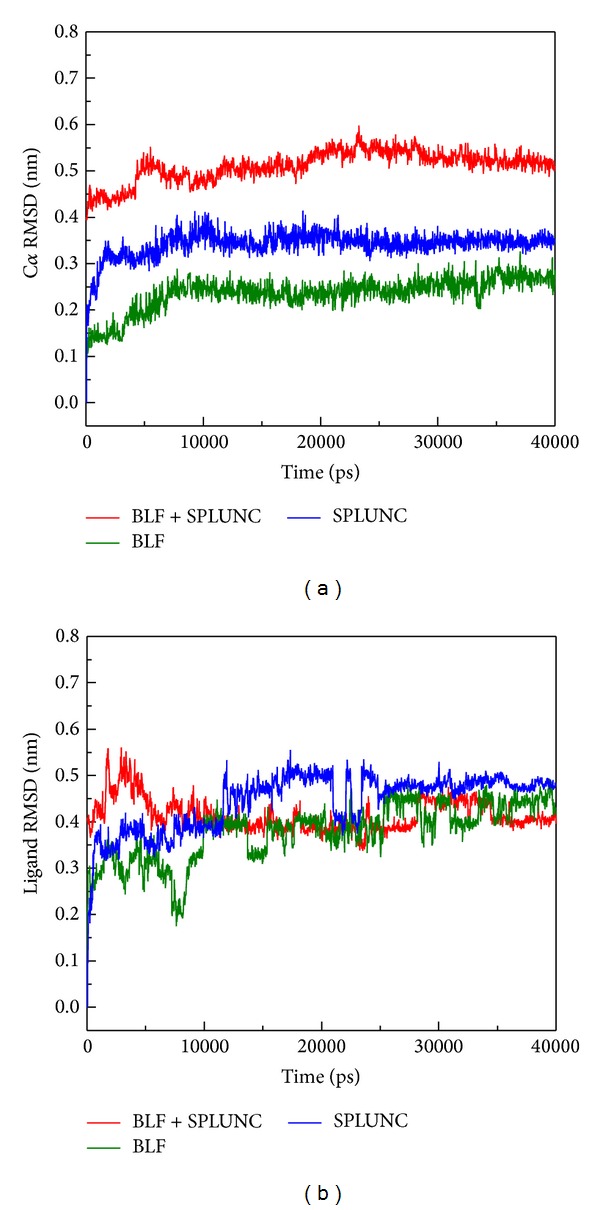
RMSD analysis for BLF, SPLUNC, and protein-protein complex (BLF and SPLUNC) among all simulation times. (a) RMSD value of the C*α* of all proteins. (b) RMAD value of lipopolysaccharide in the protein binding site.

**Figure 8:**
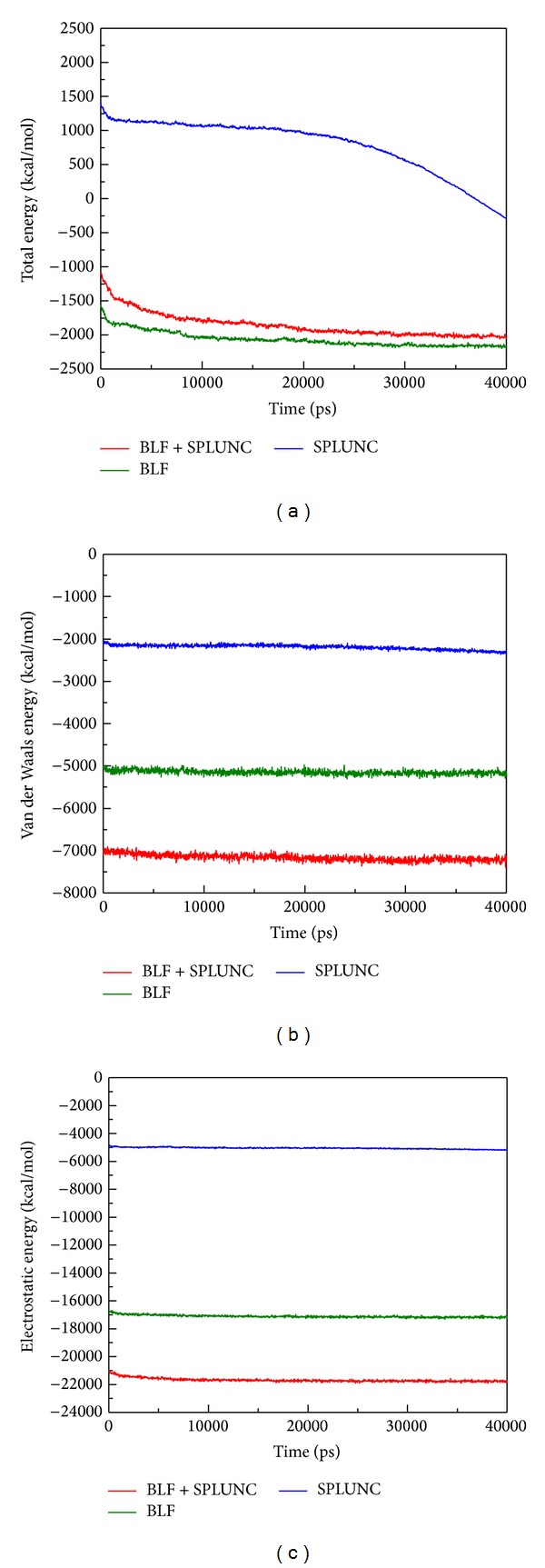
Energy analysis for BLF, SPLUNC, and protein-protein complex (BLF and SPLUNC) during 40000 ps simulation: (a) total energy, (b) Van der Waals (VDW) energy, and (c) electrostatic energy.

**Figure 9:**
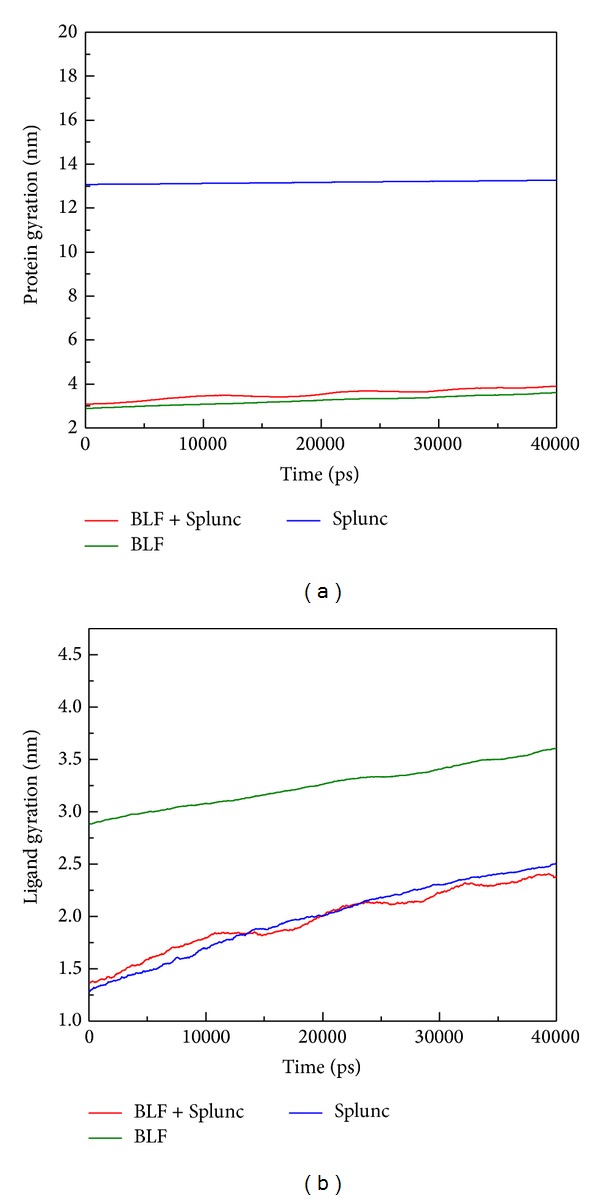
Radius of gyration analysis to measure the compactness. (a) Protein structure of BLF, SPLUNC, and protein-protein complex (BLF and SPLUNC). (b) Lipopolysaccharide.

**Figure 10:**
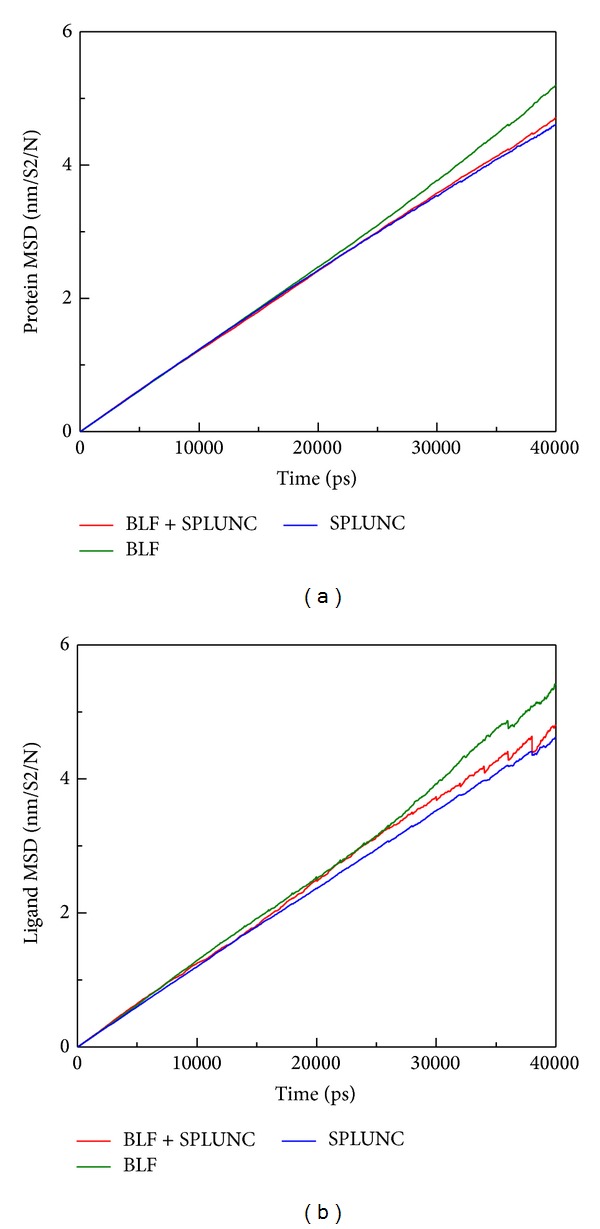
Mean square displacement (MSD) of atoms for atom migration assay. (a) Protein structure of BLF, SPLUNC, and protein-protein complex (BLF and SPLUNC). (b) Lipopolysaccharide.

Unfortunately, there have been no prior studies concerning these two as a combined effect to neutralize LPS in the related literature. Therefore we conducted this study to assist in considering possible future drug invention to intensify innate immune deficient patients in a silicon model first and simple culture based TEM survey. Based on RMSD analysis for BLF, SPLUNC, and protein-protein complex (BLF and SPLUNC) with 40 nm simulation time, we confirm that the BLF-Splunc-1-LPS combination is the most stable one (Figure 7). The energy analysis for BLF, SPLUNC, and protein-protein complex (BLF and SPLUNC) with LPS during 40000 ps simulation showed the lowest total energy, Van der Waals (VDW) energy, and electrostatic energy (Figure 8). Radius of gyration analysis (Figure 9) and mean square displacement (MSD) (Figure 10) of atoms for atom migration assay both showed stable binding of protein-protein complex (BLF and SPLUNC) to LPS even after 40 ns of simulation. The migration of BLF-Splunc-LPS complex is less than BLF alone binding with LPS. Thus, the BLF-Splunc-LPS complex is more stable than BLF-LPS complex and suggests the Splunc help the BLF binding to LPS. However, we still need studies to survey if there were any synergic effects of the BLF and Splunc.

## 5. Conclusion

Splunc-1 is considered to be a material mainly secreted from the upper airway. Lactoferricin is also considered to have a wide antimicrobial activity against bacterial, viral, and fungal pathogens. Both of them can contribute to immune function and can be found in the upper airway. We also found both of them could have stable binding to LPS related pathogens. Based on these findings it is promising to develop new drug or find new drugs and deliver them by the nasal cavity or oral spray or even orally to strengthen innate immunity and prevent comorbidities caused by pathogen related infections in the future.

## Figures and Tables

**Table 1:** Top ten ranking of docking binding affinity of Splunc-1 and lactoferricin.

Pose number	ZDOCK score
Pose 1	24.84
Pose 2	24.78
Pose 3	24.6
Pose 4	23.8
Pose 5	23.7
Pose 6	23.7
Pose 7	23.64
Pose 8	23.6
Pose 9	23.5
Pose 10	23.38
